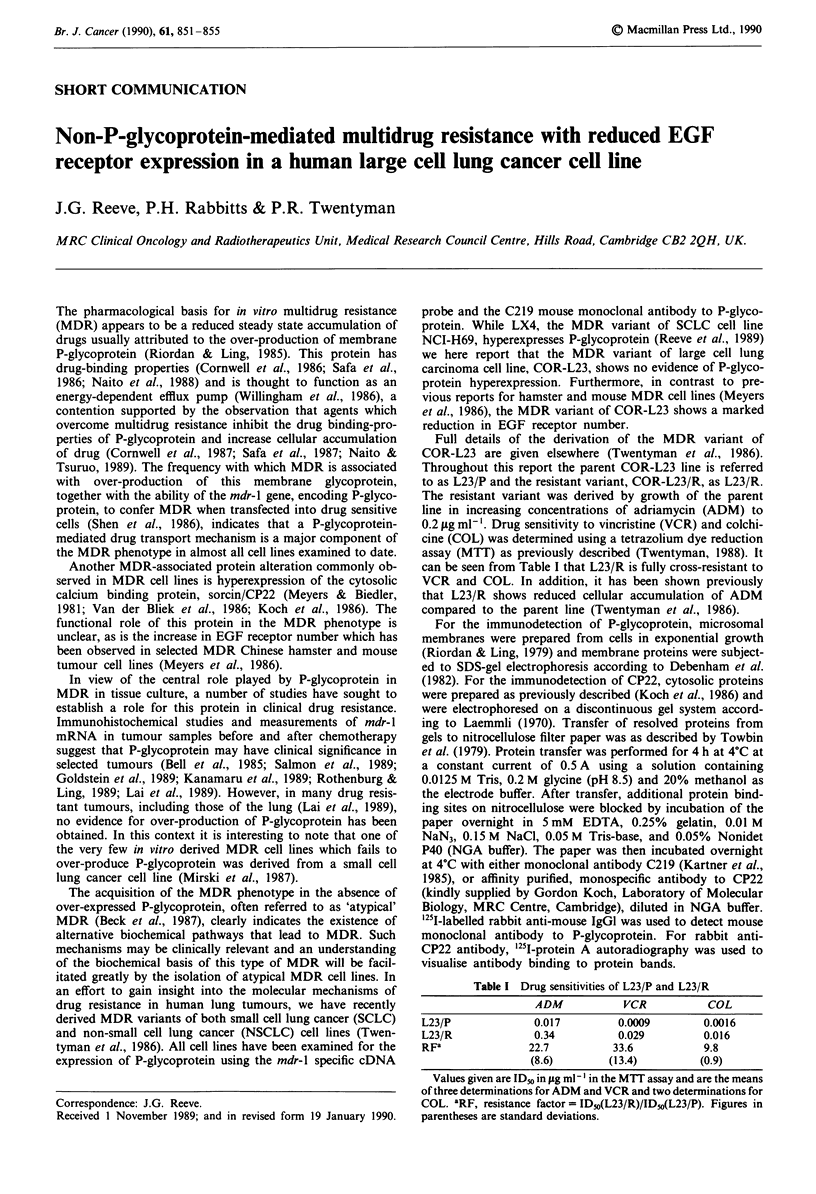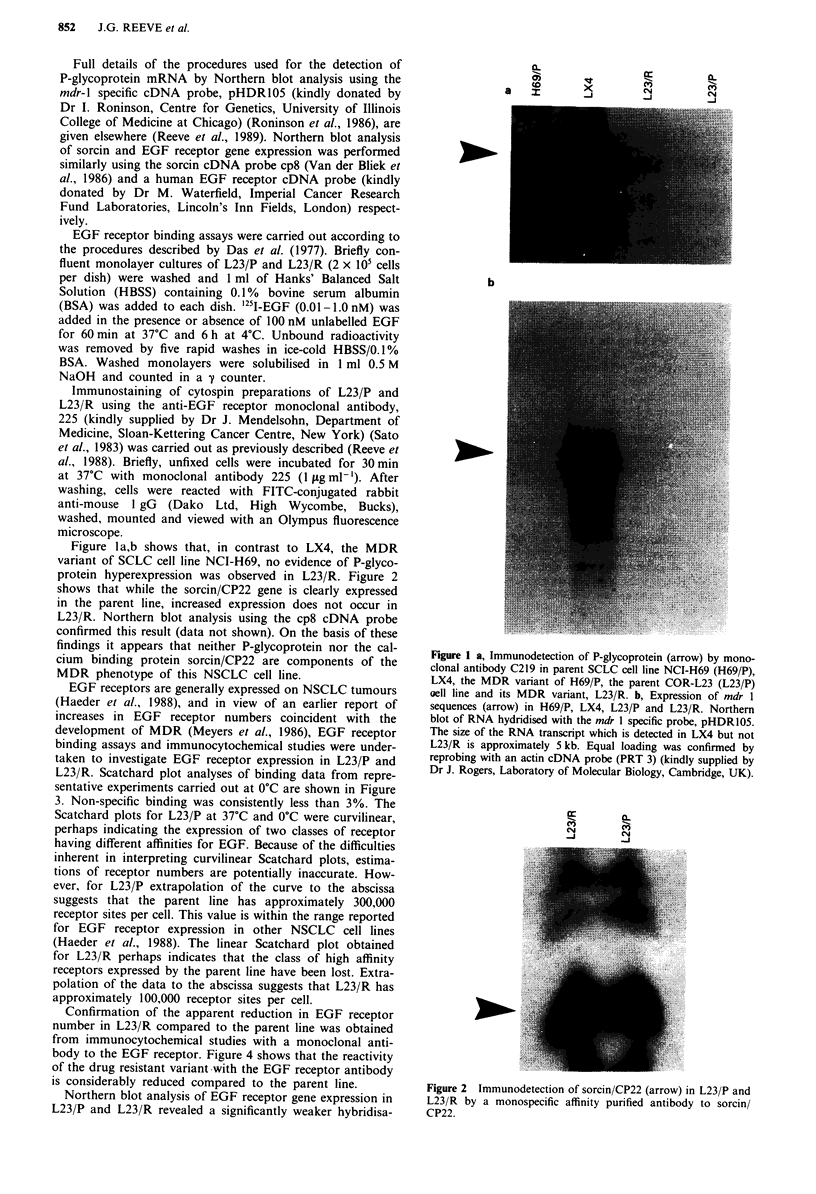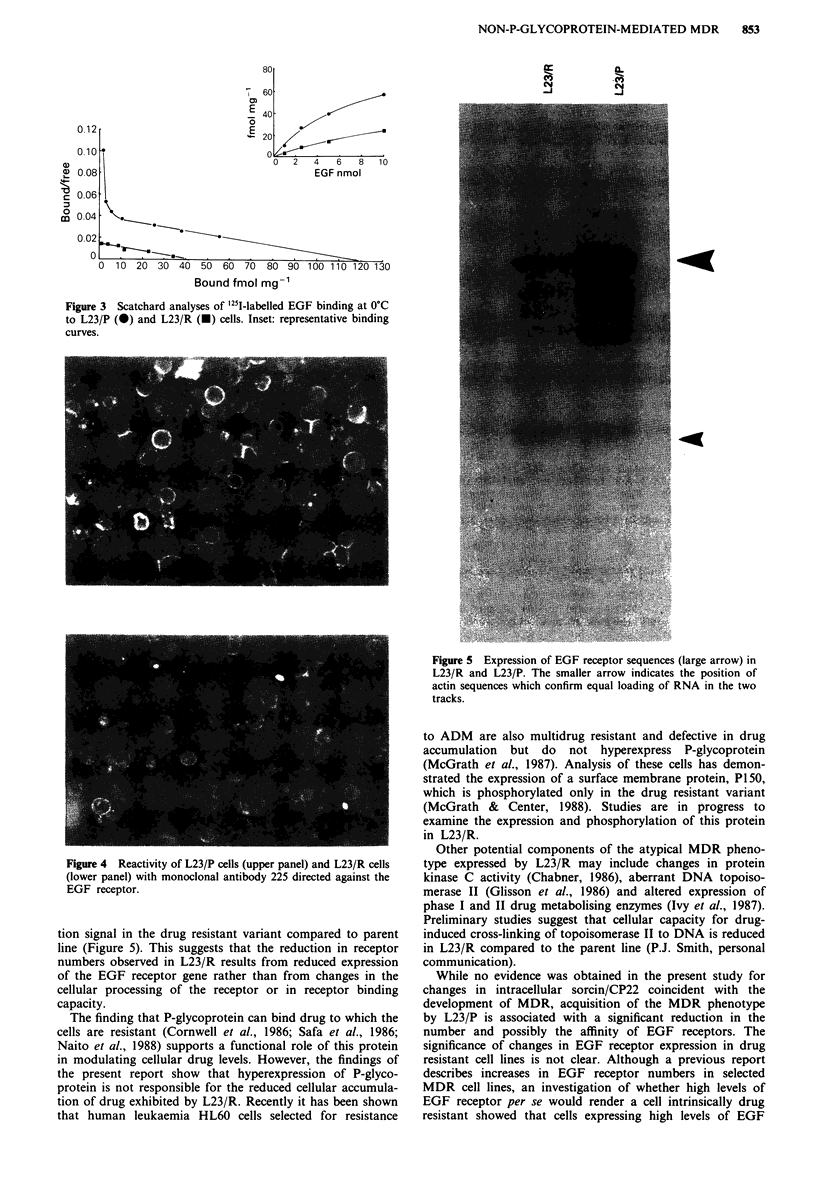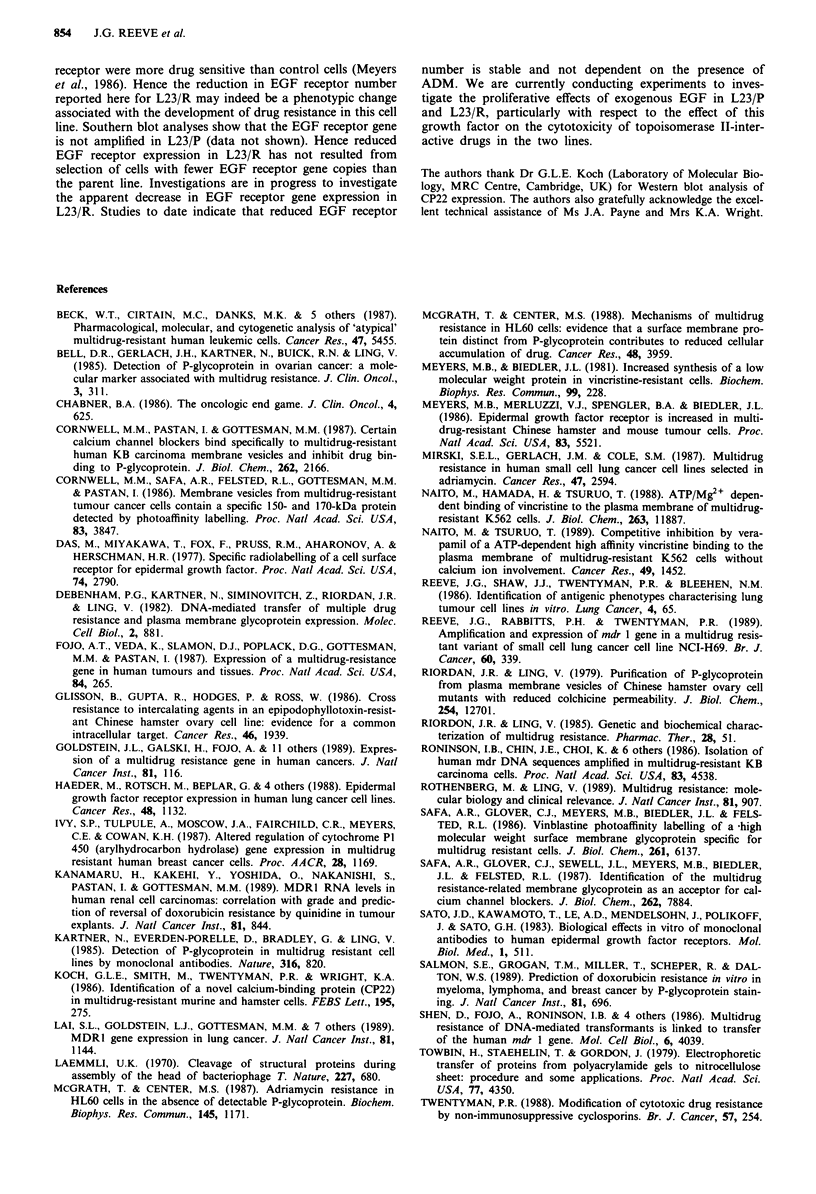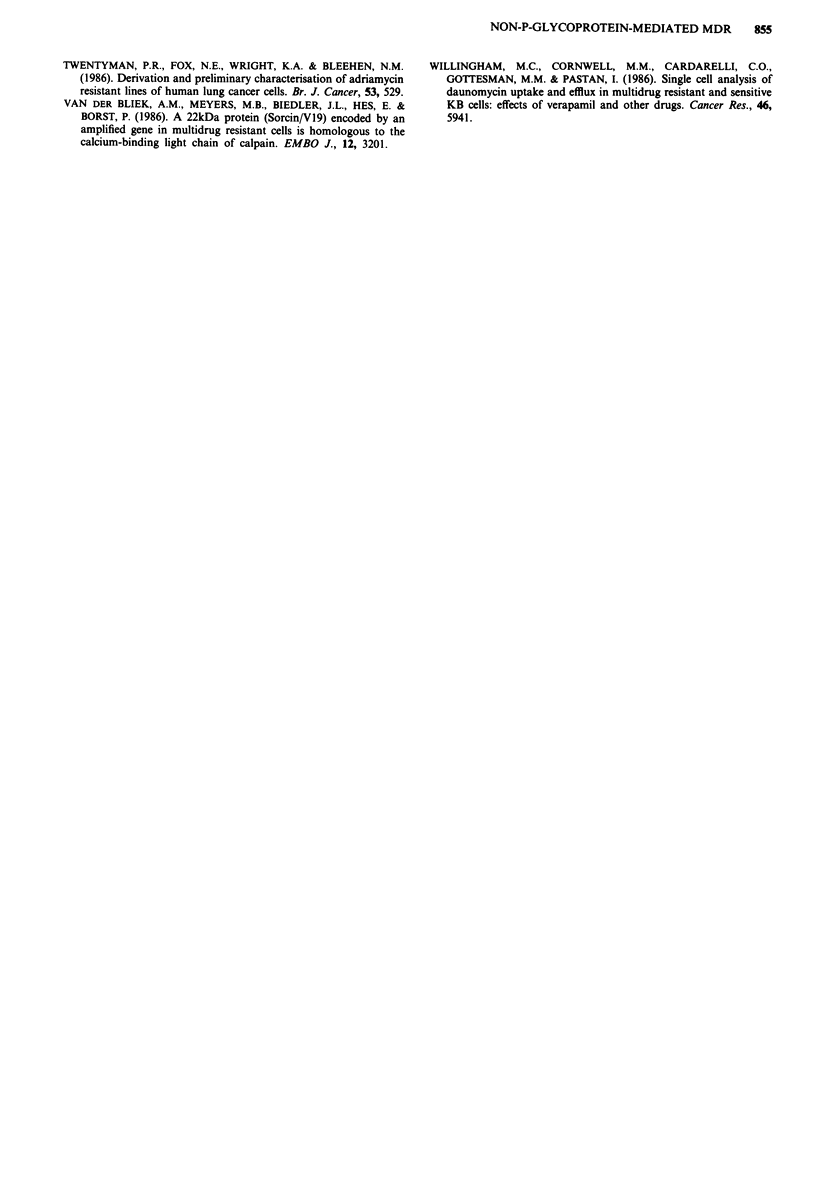# Non-P-glycoprotein-mediated multidrug resistance with reduced EGF receptor expression in a human large cell lung cancer cell line.

**DOI:** 10.1038/bjc.1990.190

**Published:** 1990-06

**Authors:** J. G. Reeve, P. H. Rabbitts, P. R. Twentyman

**Affiliations:** MRC Clinical Oncology and Radiotherapeutics Unit, Medical Research Council Centre, Cambridge, UK.

## Abstract

**Images:**


Br. J. Cancer (1990), 61, 851-855 © Macmillan Press Ltd., 1990

SHORT COMMUNICATION

### Non-P-glycoprotein-mediated multidrug resistance with reduced EGF receptor expression in a human large cell lung cancer cell line

J.G. Reeve, P.H. Rabbitts & P.R. Twentyman

*MRC Clinical Oncology and Radiotherapeutics Unit, Medical Research Council Centre, Hills Road, Cambridge CB2 2QH, UK.*

The pharmacological basis for *in vitro* multidrug resistance (MDR) appears to be a reduced steady state accumulation of drugs usually attributed to the over-production of membrane P-glycoprotein (Riordan & Ling, 1985). This protein has drug-binding properties (Cornwell *et al.*, 1986; Safa *et al.*, 1986; Naito *et al.*, 1988) and is thought to function as an energy-dependent efflux pump (Willingham *et al.*, 1986), a contention supported by the observation that agents which overcome multidrug resistance inhibit the drug binding-properties of P-glycoprotein and increase cellular accumulation of drug (Cornwell *et al.*, 1987; Safa *et al.*, 1987; Naito & Tsuruo, 1989). The frequency with which MDR is associated with over-production of this membrane glycoprotein, together with the ability of the *mdr*-1 gene, encoding P-glycoprotein, to confer MDR when transfected into drug sensitive cells (Shen *et al.*, 1986), indicates that a P-glycoprotein-mediated drug transport mechanism is a major component of the MDR phenotype in almost all cell lines examined to date.

Another MDR-associated protein alteration commonly observed in MDR cell lines is hyperexpression of the cytosolic calcium binding protein, sorcin/CP22 (Meyers & Biedler, 1981; Van der Bliek *et al.*, 1986; Koch *et al.*, 1986). The functional role of this protein in the MDR phenotype is unclear, as is the increase in EGF receptor number which has been observed in selected MDR Chinese hamster and mouse tumour cell lines (Meyers *et al.*, 1986).

In view of the central role played by P-glycoprotein in MDR in tissue culture, a number of studies have sought to establish a role for this protein in clinical drug resistance. Immunohistochemical studies and measurements of *mdr*-1 mRNA in tumour samples before and after chemotherapy suggest that P-glycoprotein may have clinical significance in selected tumours (Bell *et al.*, 1985; Salmon *et al.*, 1989; Goldstein *et al.*, 1989; Kanamaru *et al.*, 1989; Rothenburg & Ling, 1989; Lai *et al.*, 1989). However, in many drug resistant tumours, including those of the lung (Lai *et al.*, 1989), no evidence for over-production of P-glycoprotein has been obtained. In this context it is interesting to note that one of the very few *in vitro* derived MDR cell lines which fails to over-produce P-glycoprotein was derived from a small cell lung cancer cell line (Mirski *et al.*, 1987).

The acquisition of the MDR phenotype in the absence of over-expressed P-glycoprotein, often referred to as 'atypical' MDR (Beck *et al.*, 1987), clearly indicates the existence of alternative biochemical pathways that lead to MDR. Such mechanisms may be clinically relevant and an understanding of the biochemical basis of this type of MDR will be facilitated greatly by the isolation of atypical MDR cell lines. In an effort to gain insight into the molecular mechanisms of drug resistance in human lung tumours, we have recently derived MDR variants of both small cell lung cancer (SCLC) and non-small cell lung cancer (NSCLC) cell lines (Twentyman *et al.*, 1986). All cell lines have been examined for the expression of P-glycoprotein using the *mdr*-1 specific cDNA probe and the C219 mouse monoclonal antibody to P-glycoprotein. While LX4, the MDR variant of SCLC cell line NCI-H69, hyperexpresses P-glycoprotein (Reeve *et al.*, 1989) we here report that the MDR variant of large cell lung carcinoma cell line, COR-L23, shows no evidence of P-glycoprotein hyperexpression. Furthermore, in contrast to previous reports for hamster and mouse MDR cell lines (Meyers *et al.*, 1986), the MDR variant of COR-L23 shows a marked reduction in EGF receptor number.

Full details of the derivation of the MDR variant of COR-L23 are given elsewhere (Twentyman *et al.*, 1986). Throughout this report the parent COR-L23 line is referred to as L23/P and the resistant variant, COR-L23/R, as L23/R. The resistant variant was derived by growth of the parent line in increasing concentrations of adriamycin (ADM) to 0.2 $\mu g\,ml^{-1}$. Drug sensitivity to vincristine (VCR) and colchicine (COL) was determined using a tetrazolium dye reduction assay (MTT) as previously described (Twentyman, 1988). It can be seen from Table I that L23/R is fully cross-resistant to VCR and COL. In addition, it has been shown previously that L23/R shows reduced cellular accumulation of ADM compared to the parent line (Twentyman *et al.*, 1986).

For the immunodetection of P-glycoprotein, microsomal membranes were prepared from cells in exponential growth (Riordan & Ling, 1979) and membrane proteins were subjected to SDS-gel electrophoresis according to Debenham *et al.* (1982). For the immunodetection of CP22, cytosolic proteins were prepared as previously described (Koch *et al.*, 1986) and were electrophoresed on a discontinuous gel system according to Laemmli (1970). Transfer of resolved proteins from gels to nitrocellulose filter paper was as described by Towbin *et al.* (1979). Protein transfer was performed for 4 h at 4°C at a constant current of 0.5 A using a solution containing 0.0125 M Tris, 0.2 M glycine (pH 8.5) and 20% methanol as the electrode buffer. After transfer, additional protein binding sites on nitrocellulose were blocked by incubation of the paper overnight in 5 mM EDTA, 0.25% gelatin, 0.01 M $NaN_3$, 0.15 M NaCl, 0.05 M Tris-base, and 0.05% Nonidet P40 (NGA buffer). The paper was then incubated overnight at 4°C with either monoclonal antibody C219 (Kartner *et al.*, 1985), or affinity purified, monospecific antibody to CP22 (kindly supplied by Gordon Koch, Laboratory of Molecular Biology, MRC Centre, Cambridge), diluted in NGA buffer. $^{125}$I-labelled rabbit anti-mouse IgGl was used to detect mouse monoclonal antibody to P-glycoprotein. For rabbit anti-CP22 antibody, $^{125}$I-protein A autoradiography was used to visualise antibody binding to protein bands.

**Table I** Drug sensitivities of L23/P and L23/R

| | *ADM* | *VCR* | *COL* |
|---|---|---|---|
| L23/P | 0.017 | 0.0009 | 0.0016 |
| L23/R | 0.34 | 0.029 | 0.016 |
| RF[a] | 22.7 | 33.6 | 9.8 |
| | (8.6) | (13.4) | (0.9) |

Values given are $ID_{50}$ in $\mu g\,ml^{-1}$ in the MTT assay and are the means of three determinations for ADM and VCR and two determinations for COL. [a]RF, resistance factor = $ID_{50}$(L23/R)/$ID_{50}$(L23/P). Figures in parentheses are standard deviations.

Correspondence: J.G. Reeve.
Received 1 November 1989; and in revised form 19 January 1990.

Full details of the procedures used for the detection of P-glycoprotein mRNA by Northern blot analysis using the *mdr*-1 specific cDNA probe, pHDR105 (kindly donated by Dr I. Roninson, Centre for Genetics, University of Illinois College of Medicine at Chicago) (Roninson *et al.*, 1986), are given elsewhere (Reeve *et al.*, 1989). Northern blot analysis of sorcin and EGF receptor gene expression was performed similarly using the sorcin cDNA probe cp8 (Van der Bliek *et al.*, 1986) and a human EGF receptor cDNA probe (kindly donated by Dr M. Waterfield, Imperial Cancer Research Fund Laboratories, Lincoln's Inn Fields, London) respectively.

EGF receptor binding assays were carried out according to the procedures described by Das *et al.* (1977). Briefly confluent monolayer cultures of L23/P and L23/R ($2 \times 10^5$ cells per dish) were washed and 1 ml of Hanks' Balanced Salt Solution (HBSS) containing 0.1% bovine serum albumin (BSA) was added to each dish. $^{125}$I-EGF (0.01–1.0 nM) was added in the presence or absence of 100 nM unlabelled EGF for 60 min at 37°C and 6 h at 4°C. Unbound radioactivity was removed by five rapid washes in ice-cold HBSS/0.1% BSA. Washed monolayers were solubilised in 1 ml 0.5 M NaOH and counted in a $\gamma$ counter.

Immunostaining of cytospin preparations of L23/P and L23/R using the anti-EGF receptor monoclonal antibody, 225 (kindly supplied by Dr J. Mendelsohn, Department of Medicine, Sloan-Kettering Cancer Centre, New York) (Sato *et al.*, 1983) was carried out as previously described (Reeve *et al.*, 1988). Briefly, unfixed cells were incubated for 30 min at 37°C with monoclonal antibody 225 ($1\ \mu g\ ml^{-1}$). After washing, cells were reacted with FITC-conjugated rabbit anti-mouse 1 gG (Dako Ltd, High Wycombe, Bucks), washed, mounted and viewed with an Olympus fluorescence microscope.

Figure 1a,b shows that, in contrast to LX4, the MDR variant of SCLC cell line NCI-H69, no evidence of P-glycoprotein hyperexpression was observed in L23/R. Figure 2 shows that while the sorcin/CP22 gene is clearly expressed in the parent line, increased expression does not occur in L23/R. Northern blot analysis using the cp8 cDNA probe confirmed this result (data not shown). On the basis of these findings it appears that neither P-glycoprotein nor the calcium binding protein sorcin/CP22 are components of the MDR phenotype of this NSCLC cell line.

EGF receptors are generally expressed on NSCLC tumours (Haeder *et al.*, 1988), and in view of an earlier report of increases in EGF receptor numbers coincident with the development of MDR (Meyers *et al.*, 1986), EGF receptor binding assays and immunocytochemical studies were undertaken to investigate EGF receptor expression in L23/P and L23/R. Scatchard plot analyses of binding data from representative experiments carried out at 0°C are shown in Figure 3. Non-specific binding was consistently less than 3%. The Scatchard plots for L23/P at 37°C and 0°C were curvilinear, perhaps indicating the expression of two classes of receptor having different affinities for EGF. Because of the difficulties inherent in interpreting curvilinear Scatchard plots, estimations of receptor numbers are potentially inaccurate. However, for L23/P extrapolation of the curve to the abscissa suggests that the parent line has approximately 300,000 receptor sites per cell. This value is within the range reported for EGF receptor expression in other NSCLC cell lines (Haeder *et al.*, 1988). The linear Scatchard plot obtained for L23/R perhaps indicates that the class of high affinity receptors expressed by the parent line have been lost. Extrapolation of the data to the abscissa suggests that L23/R has approximately 100,000 receptor sites per cell.

Confirmation of the apparent reduction in EGF receptor number in L23/R compared to the parent line was obtained from immunocytochemical studies with a monoclonal antibody to the EGF receptor. Figure 4 shows that the reactivity of the drug resistant variant with the EGF receptor antibody is considerably reduced compared to the parent line.

Northern blot analysis of EGF receptor gene expression in L23/P and L23/R revealed a significantly weaker hybridisa-

**Figure 1** **a**, Immunodetection of P-glycoprotein (arrow) by monoclonal antibody C219 in parent SCLC cell line NCI-H69 (H69/P), LX4, the MDR variant of H69/P, the parent COR-L23 (L23/P) cell line and its MDR variant, L23/R. **b**, Expression of *mdr* 1 sequences (arrow) in H69/P, LX4, L23/P and L23/R. Northern blot of RNA hydridised with the *mdr* 1 specific probe, pHDR105. The size of the RNA transcript which is detected in LX4 but not L23/R is approximately 5 kb. Equal loading was confirmed by reprobing with an actin cDNA probe (PRT 3) (kindly supplied by Dr J. Rogers, Laboratory of Molecular Biology, Cambridge, UK).

**Figure 2** Immunodetection of sorcin/CP22 (arrow) in L23/P and L23/R by a monospecific affinity purified antibody to sorcin/CP22.

**Figure 3** Scatchard analyses of $^{125}$I-labelled EGF binding at 0°C to L23/P (●) and L23/R (■) cells. Inset: representative binding curves.

**Figure 4** Reactivity of L23/P cells (upper panel) and L23/R cells (lower panel) with monoclonal antibody 225 directed against the EGF receptor.

tion signal in the drug resistant variant compared to parent line (Figure 5). This suggests that the reduction in receptor numbers observed in L23/R results from reduced expression of the EGF receptor gene rather than from changes in the cellular processing of the receptor or in receptor binding capacity.

The finding that P-glycoprotein can bind drug to which the cells are resistant (Cornwell *et al.*, 1986; Safa *et al.*, 1986; Naito *et al.*, 1988) supports a functional role of this protein in modulating cellular drug levels. However, the findings of the present report show that hyperexpression of P-glycoprotein is not responsible for the reduced cellular accumulation of drug exhibited by L23/R. Recently it has been shown that human leukaemia HL60 cells selected for resistance

**Figure 5** Expression of EGF receptor sequences (large arrow) in L23/R and L23/P. The smaller arrow indicates the position of actin sequences which confirm equal loading of RNA in the two tracks.

to ADM are also multidrug resistant and defective in drug accumulation but do not hyperexpress P-glycoprotein (McGrath *et al.*, 1987). Analysis of these cells has demonstrated the expression of a surface membrane protein, P150, which is phosphorylated only in the drug resistant variant (McGrath & Center, 1988). Studies are in progress to examine the expression and phosphorylation of this protein in L23/R.

Other potential components of the atypical MDR phenotype expressed by L23/R may include changes in protein kinase C activity (Chabner, 1986), aberrant DNA topoisomerase II (Glisson *et al.*, 1986) and altered expression of phase I and II drug metabolising enzymes (Ivy *et al.*, 1987). Preliminary studies suggest that cellular capacity for drug-induced cross-linking of topoisomerase II to DNA is reduced in L23/R compared to the parent line (P.J. Smith, personal communication).

While no evidence was obtained in the present study for changes in intracellular sorcin/CP22 coincident with the development of MDR, acquisition of the MDR phenotype by L23/P is associated with a significant reduction in the number and possibly the affinity of EGF receptors. The significance of changes in EGF receptor expression in drug resistant cell lines is not clear. Although a previous report describes increases in EGF receptor numbers in selected MDR cell lines, an investigation of whether high levels of EGF receptor *per se* would render a cell intrinsically drug resistant showed that cells expressing high levels of EGF

receptor were more drug sensitive than control cells (Meyers et al., 1986). Hence the reduction in EGF receptor number reported here for L23/R may indeed be a phenotypic change associated with the development of drug resistance in this cell line. Southern blot analyses show that the EGF receptor gene is not amplified in L23/P (data not shown). Hence reduced EGF receptor expression in L23/R has not resulted from selection of cells with fewer EGF receptor gene copies than the parent line. Investigations are in progress to investigate the apparent decrease in EGF receptor gene expression in L23/R. Studies to date indicate that reduced EGF receptor number is stable and not dependent on the presence of ADM. We are currently conducting experiments to investigate the proliferative effects of exogenous EGF in L23/P and L23/R, particularly with respect to the effect of this growth factor on the cytotoxicity of topoisomerase II-interactive drugs in the two lines.

The authors thank Dr G.L.E. Koch (Laboratory of Molecular Biology, MRC Centre, Cambridge, UK) for Western blot analysis of CP22 expression. The authors also gratefully acknowledge the excellent technical assistance of Ms J.A. Payne and Mrs K.A. Wright.

### References

BECK, W.T., CIRTAIN, M.C., DANKS, M.K. & 5 others (1987). Pharmacological, molecular, and cytogenetic analysis of 'atypical' multidrug-resistant human leukemic cells. *Cancer Res.*, **47**, 5455.

BELL, D.R., GERLACH, J.H., KARTNER, N., BUICK, R.N. & LING, V. (1985). Detection of P-glycoprotein in ovarian cancer: a molecular marker associated with multidrug resistance. *J. Clin. Oncol.*, **3**, 311.

CHABNER, B.A. (1986). The oncologic end game. *J. Clin. Oncol.*, **4**, 625.

CORNWELL, M.M., PASTAN, I. & GOTTESMAN, M.M. (1987). Certain calcium channel blockers bind specifically to multidrug-resistant human KB carcinoma membrane vesicles and inhibit drug binding to P-glycoprotein. *J. Biol. Chem.*, **262**, 2166.

CORNWELL, M.M., SAFA, A.R., FELSTED, R.L., GOTTESMAN, M.M. & PASTAN, I. (1986). Membrane vesicles from multidrug-resistant tumour cancer cells contain a specific 150- and 170-kDa protein detected by photoaffinity labelling. *Proc. Natl Acad. Sci. USA*, **83**, 3847.

DAS, M., MIYAKAWA, T., FOX, F., PRUSS, R.M., AHARONOV, A. & HERSCHMAN, H.R. (1977). Specific radiolabelling of a cell surface receptor for epidermal growth factor. *Proc. Natl Acad. Sci. USA*, **74**, 2790.

DEBENHAM, P.G., KARTNER, N., SIMINOVITCH, Z., RIORDAN, J.R. & LING, V. (1982). DNA-mediated transfer of multiple drug resistance and plasma membrane glycoprotein expression. *Molec. Cell Biol.*, **2**, 881.

FOJO, A.T., VEDA, K., SLAMON, D.J., POPLACK, D.G., GOTTESMAN, M.M. & PASTAN, I. (1987). Expression of a multidrug-resistance gene in human tumours and tissues. *Proc. Natl Acad. Sci. USA*, **84**, 265.

GLISSON, B., GUPTA, R., HODGES, P. & ROSS, W. (1986). Cross resistance to intercalating agents in an epipodophyllotoxin-resistant Chinese hamster ovary cell line: evidence for a common intracellular target. *Cancer Res.*, **46**, 1939.

GOLDSTEIN, J.L., GALSKI, H., FOJO, A. & 11 others (1989). Expression of a multidrug resistance gene in human cancers. *J. Natl Cancer Inst.*, **81**, 116.

HAEDER, M., ROTSCH, M., BEPLAR, G. & 4 others (1988). Epidermal growth factor receptor expression in human lung cancer cell lines. *Cancer Res.*, **48**, 1132.

IVY, S.P., TULPULE, A., MOSCOW, J.A., FAIRCHILD, C.R., MEYERS, C.E. & COWAN, K.H. (1987). Altered regulation of cytochrome P1 450 (arylhydrocarbon hydrolase) gene expression in multidrug resistant human breast cancer cells. *Proc. AACR*, **28**, 1169.

KANAMARU, H., KAKEHI, Y., YOSHIDA, O., NAKANISHI, S., PASTAN, I. & GOTTESMAN, M.M. (1989). MDR1 RNA levels in human renal cell carcinomas: correlation with grade and prediction of reversal of doxorubicin resistance by quinidine in tumour explants. *J. Natl Cancer Inst.*, **81**, 844.

KARTNER, N., EVERDEN-PORELLE, D., BRADLEY, G. & LING, V. (1985). Detection of P-glycoprotein in multidrug resistant cell lines by monoclonal antibodies. *Nature*, **316**, 820.

KOCH, G.L.E., SMITH, M., TWENTYMAN, P.R. & WRIGHT, K.A. (1986). Identification of a novel calcium-binding protein (CP22) in multidrug-resistant murine and hamster cells. *FEBS Lett.*, **195**, 275.

LAI, S.L., GOLDSTEIN, L.J., GOTTESMAN, M.M. & 7 others (1989). MDR1 gene expression in lung cancer. *J. Natl Cancer Inst.*, **81**, 1144.

LAEMMLI, U.K. (1970). Cleavage of structural proteins during assembly of the head of bacteriophage *T. Nature*, **227**, 680.

MCGRATH, T. & CENTER, M.S (1987). Adriamycin resistance in HL60 cells in the absence of detectable P-glycoprotein. *Biochem. Biophys. Res. Commun.*, **145**, 1171.

MCGRATH, T. & CENTER, M.S. (1988). Mechanisms of multidrug resistance in HL60 cells: evidence that a surface membrane protein distinct from P-glycoprotein contributes to reduced cellular accumulation of drug. *Cancer Res.*, **48**, 3959.

MEYERS, M.B., & BIEDLER, J.L. (1981). Increased synthesis of a low molecular weight protein in vincristine-resistant cells. *Biochem. Biophys. Res. Commun.*, **99**, 228.

MEYERS, M.B., MERLUZZI, V.J., SPENGLER, B.A. & BIEDLER, J.L. (1986). Epidermal growth factor receptor is increased in multidrug-resistant Chinese hamster and mouse tumour cells. *Proc. Natl Acad. Sci. USA*, **83**, 5521.

MIRSKI, S.E.L., GERLACH, J.M. & COLE, S.M. (1987). Multidrug resistance in human small cell lung cancer cell lines selected in adriamycin. *Cancer Res.*, **47**, 2594.

NAITO, M., HAMADA, H. & TSURUO, T. (1988). ATP/$Mg^{2+}$ dependent binding of vincristine to the plasma membrane of multidrug-resistant K562 cells. *J. Biol. Chem.*, **263**, 11887.

NAITO, M. & TSURUO, T. (1989). Competitive inhibition by verapamil of a ATP-dependent high affinity vincristine binding to the plasma membrane of multidrug-resistant K562 cells without calcium ion involvement. *Cancer Res.*, **49**, 1452.

REEVE, J.G., SHAW, J.J., TWENTYMAN, P.R. & BLEEHEN, N.M. (1986). Identification of antigenic phenotypes characterising lung tumour cell lines *in vitro*. *Lung Cancer*, **4**, 65.

REEVE, J.G., RABBITTS, P.H. & TWENTYMAN, P.R. (1989). Amplification and expression of *mdr* 1 gene in a multidrug resistant variant of small cell lung cancer cell line NCI-H69. *Br. J. Cancer*, **60**, 339.

RIORDAN, J.R. & LING, V. (1979). Purification of P-glycoprotein from plasma membrane vesicles of Chinese hamster ovary cell mutants with reduced colchicine permeability. *J. Biol. Chem.*, **254**, 12701.

RIORDON, J.R. & LING, V. (1985). Genetic and biochemical characterization of multidrug resistance. *Pharmac. Ther.*, **28**, 51.

RONINSON, I.B., CHIN, J.E., CHOI, K. & 6 others (1986). Isolation of human mdr DNA sequences amplified in multidrug-resistant KB carcinoma cells. *Proc. Natl Acad. Sci. USA*, **83**, 4538.

ROTHENBERG, M. & LING, V. (1989). Multidrug resistance: molecular biology and clinical relevance. *J. Natl Cancer Inst.*, **81**, 907.

SAFA, A.R., GLOVER, C.J., MEYERS, M.B., BIEDLER, J.L. & FELSTED, R.L. (1986). Vinblastine photoaffinity labelling of a high molecular weight surface membrane glycoprotein specific for multidrug resistant cells. *J. Biol. Chem.*, **261**, 6137.

SAFA, A.R., GLOVER, C.J., SEWELL, J.L., MEYERS, M.B., BIEDLER, J.L. & FELSTED, R.L. (1987). Identification of the multidrug resistance-related membrane glycoprotein as an acceptor for calcium channel blockers. *J. Biol. Chem.*, **262**, 7884.

SATO, J.D., KAWAMOTO, T., LE, A.D., MENDELSOHN, J., POLIKOFF, J. & SATO, G.H. (1983). Biological effects in vitro of monoclonal antibodies to human epidermal growth factor receptors. *Mol. Biol. Med.*, **1**, 511.

SALMON, S.E., GROGAN, T.M., MILLER, T., SCHEPER, R. & DALTON, W.S. (1989). Prediction of doxorubicin resistance *in vitro* in myeloma, lymphoma, and breast cancer by P-glycoprotein staining. *J. Natl Cancer Inst.*, **81**, 696.

SHEN, D., FOJO, A., RONINSON, I.B. & 4 others (1986). Multidrug resistance of DNA-mediated transformants is linked to transfer of the human *mdr* 1 gene. *Mol. Cell Biol.*, **6**, 4039.

TOWBIN, H., STAEHELIN, T. & GORDON, J. (1979). Electrophoretic transfer of proteins from polyacrylamide gels to nitrocellulose sheet: procedure and some applications. *Proc. Natl Acad. Sci. USA*, **77**, 4350.

TWENTYMAN, P.R. (1988). Modification of cytotoxic drug resistance by non-immunosuppressive cyclosporins. *Br. J. Cancer*, **57**, 254.

TWENTYMAN, P.R., FOX, N.E., WRIGHT, K.A. & BLEEHEN, N.M. (1986). Derivation and preliminary characterisation of adriamycin resistant lines of human lung cancer cells. *Br. J. Cancer*, **53**, 529.

VAN DER BLIEK, A.M., MEYERS, M.B., BIEDLER, J.L., HES, E. & BORST, P. (1986). A 22kDa protein (Sorcin/V19) encoded by an amplified gene in multidrug resistant cells is homologous to the calcium-binding light chain of calpain. *EMBO J.*, **12**, 3201.

WILLINGHAM, M.C., CORNWELL, M.M., CARDARELLI, C.O., GOTTESMAN, M.M. & PASTAN, I. (1986). Single cell analysis of daunomycin uptake and efflux in multidrug resistant and sensitive KB cells: effects of verapamil and other drugs. *Cancer Res.*, **46**, 5941.